# Environmental Samples Test Negative for Avian Influenza Virus H5N1 Four Months after Mass Mortality at A Seabird Colony

**DOI:** 10.3390/pathogens12040584

**Published:** 2023-04-12

**Authors:** Robert W. Furness, Sheila C. Gear, Kees C. J. Camphuysen, Glen Tyler, Dilhani de Silva, Caroline J. Warren, Joe James, Scott M. Reid, Ashley C. Banyard

**Affiliations:** 1 MacArthur Green, 95 South Woodside Road, Glasgow G20 6NT, UK; 2 School of Biodiversity, One Health and Veterinary Medicine, University of Glasgow, Glasgow G12 8QQ, UK; 3 Foula Ranger Service, Magdala, Foula, Shetland ZE2 9PN, UK; sheilagear9@gmail.com; 4 Royal Netherlands Institute for Sea Research, 1790 Den Burg, The Netherlands; kees.camphuysen@nioz.nl; 5 NatureScot, Lerwick, Shetland ZE1 0LL, UK; glen.tyler@nature.scot; 6 Animal and Plant Health Agency, Weybridge, Surrey KT15 3NB, UK; dilhani.desilva@apha.gov.uk (D.d.S.); caroline.warren@apha.gov.uk (C.J.W.); joe.james@apha.gov.uk (J.J.); scott.reid@apha.gov.uk (S.M.R.)

**Keywords:** highly pathogenic avian influenza, H5N1, environmental contamination, water sample, nucleic acid detection

## Abstract

High pathogenicity avian influenza (HPAI) profoundly impacted several seabird populations during the summers of 2021 and 2022. Infection spread rapidly across colonies, causing unprecedented mortality. At Foula, Shetland, 1500 breeding adult great skuas *Stercorarius skua*, totalling about two tonnes of decomposing virus-laden material, died at the colony in May−July 2022. Carcasses were left where they died as Government policy was not to remove dead birds. The factors influencing risk of further spread of infection are uncertain, but evidence suggests that HPAI can persist in water for many months in cool conditions and may be a major transmission factor for birds living in wetlands. We investigated risk of further spread of infection from water samples collected from under 45 decomposing carcasses and in three freshwater lochs/streams by sampling water in October 2022, by which time the great skua carcasses had rotted to bones, skin, and feathers. No viral genetic material was detected four months after the mortality, suggesting a low risk of seabird infection from the local environment when the seabirds would return the next breeding season. These findings, although based on a relatively small number of water samples, suggest that the high rainfall typical at Shetland probably washed away the virus from the decomposing carcasses. However, limitations to our study need to be taken on board in the design of environmental monitoring at seabird colonies during and immediately after future outbreaks of HPAI.

## 1. Introduction

An outbreak of H5N1 high pathogenicity avian influenza virus (HPAIV) killed many breeding great skuas (*Stercorarius skua*) at several colonies in Scotland during the summer of 2021 [1]. Great skuas at colonies throughout the North Atlantic were affected in 2022 even more severely. Infection during 2022 signalled a significant change in virus dynamics, with unprecedented mortalities across North Atlantic colonial seabirds of several species [2]. Over 1500 adult great skuas were found dead in May−August 2022 at Foula, Shetland—the world’s largest colony of this rare seabird [3]. As in 2021, all dead great skuas swabbed in 2022 tested positive for H5N1 virus at numerous colonies in Scotland, including Foula. Dead great skuas at Foula also showed the typical symptoms of HPAI; severe hyperextension and spasticity in the head and neck (opisthotonos) [3]. Great skuas are opportunistic scavengers and might acquire HPAIV by feeding on infected carcasses of other birds [1,2]. Scavenging behaviour has been observed in areas with high mortalities of birds, including great skuas feeding on dead northern gannets (*Morus bassanus*); however, their scavenging habits appear to be insufficient to explain the rapid spread of HPAI over the entire colony, as most scavenging occurs at sea rather than at the colony, and great skuas very rarely scavenge from dead adult great skuas. However, the predation of possibly infectious, unattended eggs and chicks, belonging to dead or dying parents, may have been an additional source of the spread of the virus through the colony from the second week of May onwards. Very few abandoned eggs and chicks were observed, despite the large number of dead adults. They simply disappeared, so they were presumably taken by opportunistic scavengers.

Apart from through scavenging and predation, avian influenza virus (AIV) might be carried to breeding colonies by individual infected great skuas returning from their winter quarters (which are from southern Europe to west Africa), or the virus might persist in freshwater environments at the colony and infect birds when they arrive back at the colony in spring [3]. Influenza viruses deposited into freshwater environments by wild birds may lead to further infections [4,5]. In situ experiments in Alaska have indicated that AIV may persist for many months in freshwater under some environmental conditions [4]. The temperature at Shetland is similar during the autumn and winter to the location where the in situ experiment was carried out, so there seemed likely to be a risk of HPAIV remaining in the environment at Shetland from one seabird breeding season to the next. Because breeding great skuas are highly territorial while breeding, birds do not normally enter the territory of neighbours, which are spaced about 20 to 150 m apart across the island. However, adult great skuas bathe communally in traditional freshwater sites [6], a habit that could also increase the risk of acquiring infection from the virus in water [7]. If one or more great skuas return to the colony at the start of the breeding season with HPAIV, the spread of infection within the colony may be through direct contact between infected and uninfected birds or through aerosol transmission of the virus from coughing, especially when these birds gather to bathe communally. The relative risk from these different pathways is unclear, but has implications in relation to possible disease control measures. There is thus a need to understand how HPAIV is carried to seabird colonies, how it spreads within colonies, and whether the virus remains within the environment of the colony from one seabird breeding season to the next, and so could re-infect birds returning to the colony each spring. The aim of this study was to assess whether HPAIV persists in water in the environment at a seabird colony from one seabird breeding season to the next, as this has never been tested. Based on previous studies [4,5] we expected to find the virus still present for some months after the mass mortality during the breeding season, but were uncertain whether it would remain throughout the winter into the next spring.

## 2. Materials and Methods

The island of Foula, 28 km west of the mainland of Shetland, is the most remote inhabited island in the British Isles, with about 25 people permanently residing there. With no shops, no cell phone coverage, and very few facilities for tourists, as well as travel constraints often imposed by the weather, the island receives few visitors, despite having spectacular scenery and wildlife. It is a small island (12 km^2^) mostly surrounded by sea cliffs of up to 370 m. Inland, the island is almost entirely covered by actively growing peat, with extensive blanket bog. The island holds some of the largest seabird colonies in the British Isles. There are domestic sheep and ponies that range across the island, as well as introduced populations of domestic dogs and cats, frogs (*Rana temporaria*), hedgehogs (*Erinaceous europaeus*), house mice (*Mus musculus*), and an endemic subspecies of field mouse (*Apodemus sylvaticus thuleo*) [8,9]. We used published data on breeding numbers of seabirds [10,11] and our own counts of birds present in October 2022 to place the results of virus testing into the context of wildlife populations that might be affected by HPAIV. We also searched for dead birds in October 2022 and from then until March 2023 as further context.

**Figure 1:**
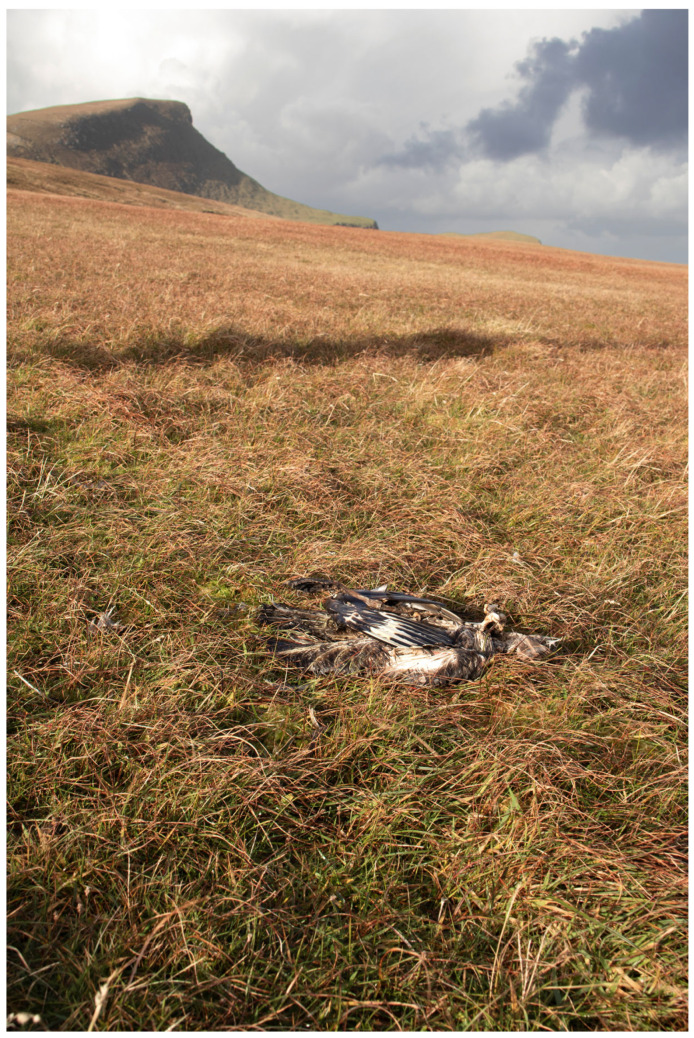
Example of a great skua carcass at Foula, Shetland, in October 2022 from immediately under which a water sample was collected for virus testing. The vegetation on which the carcasses were lying is growing in peat, with large amounts of Sphagnum moss under the grass, so that sampling water was possible from almost all of the carcasses.

**Figure 2:**
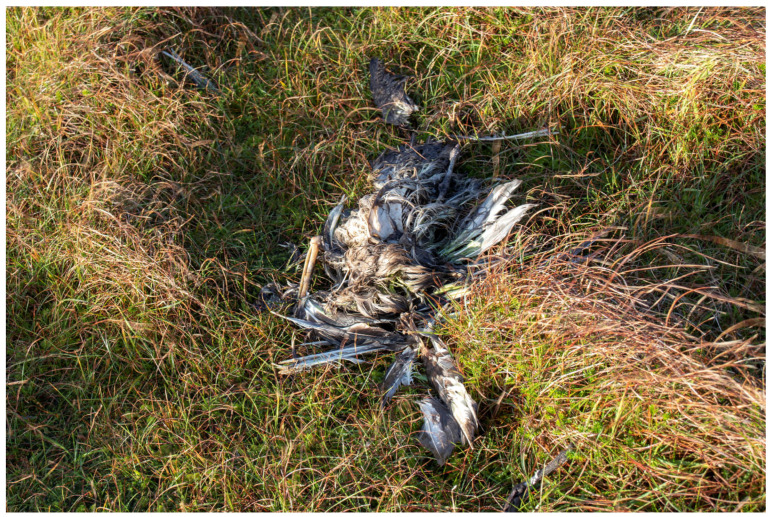
Example of a great skua carcass at Foula, Shetland, in October 2022 from immediately under which a water sample was collected from Sphagnum moss growing under the grass for virus testing.

The entire great skua colony at Foula was systematically searched five times from May until early August by K.C.J.C. and S.C.G. Locations of dead adult great skuas were mapped from GPS coordinates obtained in the field using a Garmin Etrex 32X. Primaries of both wings of each dead bird were cut to avoid any double-counting of carcasses. On 16 October 2022, four months after the mass mortality of breeding birds, we collected samples of water in Sphagnum moss from immediately beneath 45 decomposing great skuas at Foula, Shetland. The samples were only taken from under birds that appeared to have remained in the same position since death. The 4-month old carcasses had all decomposed down to the skin, bones, and loose feathers (Figure 1 and Figure 2). Fifteen of these samples were tested individually. To limit numbers to be analysed, we made two pooled samples from the other 30 birds (15 in each). We also collected water from three freshwater sites likely to be particularly at risk of harbouring HPAIV from decomposing carcasses of great skuas; two were lochs in which great skuas bathe and which contained many dead carcasses (Mill Loch and Rossies Loch) and the third was Ham Burn, which carries runoff water from much of the colony into the sea. The catchment area that feeds Ham Burn held over 1000 of the dead great skuas and so, although the sampling site was downstream from the locations of the dead birds, virus washed off the ground would be carried in that stream to the sea; thus, that sampling site was chosen to provide water drained from the ground on which most of the dead birds were located.

Previous studies have shown that virus survival in water decreases with the increase in water temperature and is lower in unfiltered water [12,13]. Avian influenza virus remained infective in water for several weeks at 10 °C [13] and for >60 days in filtered freshwater at 4 °C [12]; so, we ensured that the water samples were filtered, kept cool, and were transported to the testing laboratory as quickly as possible. Within minutes of collection, all of the water samples were filtered and then put into labelled 1.5 mL plastic tubes and chilled to 2 °C in a refrigerator. After chilling, they were put into a thermos flask and sent by guaranteed 24-h air freight delivery to the laboratory for testing. The 20 samples were tested within 48 h from the time of collection, at the Animal and Plant Health Agency (APHA) International Reference Laboratory for Avian Influenza using molecular diagnostic tools including a generic influenza. Following RNA extraction, a virus screening real-time reverse transcription polymerase chain reaction (RRT-PCR) assay targeting the matrix (M) gene [14], an RRT-PCR that detects H5 HPAI virus (HPAIV) RNA [15] and an RRT-PCR that detects N1 AIV RNA [16]. We planned to repeat water sampling every month through the winter to determine how long the virus remained in the colony.

## 3. Results and Discussion

**Figure 3:**
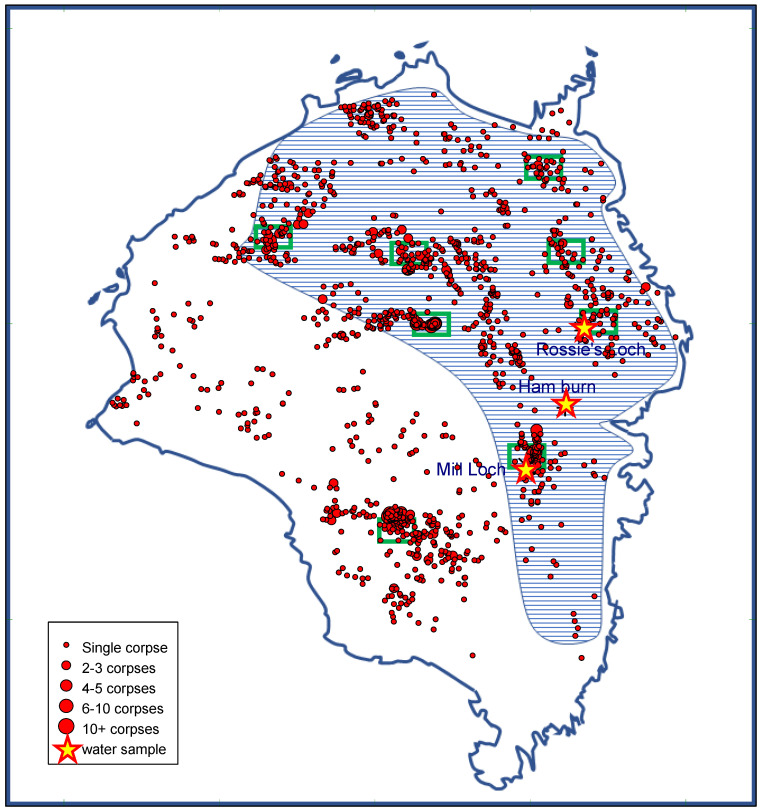
Locations of carcasses of adult great skuas found at Foula in May–August 2022, and location of water samples that were sampled on the island in October 2022. Samples from under 45 great skua carcasses were collected from throughout the blue hatched area. Samples from water bodies were from Rossies Loch, Mill Loch, and Ham Burn at points marked with stars. Green rectangles indicate locations of major bathing sites used by adult great skuas during the breeding season.

Adult great skuas found dead at colonies in several different regions of Scotland in 2022, including Foula, Shetland, tested positive for HPAIV H5N1 (Table 1). The amount of virus present was moderate in most cases (Ct scores predominantly of 20 to 30). The three great skuas from Foula that were tested in July 2022 had Ct scores of 22, 23, and 25, each. Of the 1500 adult great skuas found dead at Foula in 2022, 112 carried leg-bands (metal leg rings). Almost all of these had been banded as chicks, so they were of an exactly-known age. All of them had been banded at Shetland. The ages of these birds were from 6 to 36 years old, so all of them were of breeding age. Great skua breeding territories are distributed across most of Foula, so if infections were transmitted between breeding birds on neighbouring territories we would expect clusters of dead adults all across the colony. While there were some clusters scattered across the colony, suggesting a possible transmission process between neighbouring breeding birds, the distribution of dead adult great skuas (Figure 3) showed a tendency for carcasses to be particularly numerous in the vicinity of traditional communal club or bathing sites (these areas are marked in Figure 3 by green rectangles), suggesting the possibility that virus transmission may be particularly linked to this habit and habitat.

In the most recent counts of the breeding numbers of seabirds at Foula, there were 10,253 pairs of northern fulmars (*Fulmarus glacialis*) in 2021, a minimum of 5289 individual common guillemots (*Uria aalge*) in 2021, 6351 individual Atlantic puffins (*Fratercula arctica*) in 2016, 2443 pairs of northern gannets in 2021, 1846 pairs of great skuas in 2015, a minimum of 474 individual razorbills (*Alca torda*) in 2021, 425 pairs of black-legged kittiwakes (*Rissa tridactyla*) in 2021, 324 pairs of European shags (*Phalacrocorax aristotelis*) in 2018, 172 individual black guillemots (*Cepphus grylle*) in 2022, 100 common eiders (*Somateria mollissima*) in 2022, 21 pairs of Arctic skua (*Stercorarius parasiticus*) in 2022, 19 pairs of Arctic tern (*Sterna paradisaea*) in 2018, 13 pairs of red-throated divers (*Gavia stellata*) in 2022, 7 pairs of herring gull (*Larus argentatus*) in 2017, 4 pairs of great black-backed gull (Larus marinus) in 2021, 2 pairs of common gull (*Larus canus*) in 2017, and 1 pair of lesser black-backed gulls (*Larus fuscus*) in 2017 [10,11]. Remarkably, most of these seabirds avoided HPAIV infection, with only great skuas, northern gannets, and one or two gulls showing symptoms in the summer of 2022 [3].

In mid-October 2022, to maximize the chance of detecting HPAIV that remained in the colony after the seabirds had died or left, water samples were collected from immediately underneath the great skua carcasses across a large part of the colony (Figure 3). However, all of the samples tested negative for the presence of influenza virus RNA by all three tests. The absence of detectable viral RNA in the samples suggests that it is unlikely that water present at the sampled sites contained viral material of an infectious, or even non-infectious nature. The limit of detection of PCRs was equivalent to 10.96 EID_50_/mL of infectious virus based on comparison using a decimal dilution of an RNA standard curve of an H5N1 virus representative of the 2021/2022 epizootic. This is below the minimum infectious dose required to establish robust infection in ducks experimentally infected with representative H5N1 HPAIVs from the current epizootic (APHA, unpublished data). As such, it is reasonable to assume that birds returning to the colony in the spring would not be infected from the virus in the freshwater at this colony.

During mid-October 2022, we counted about 200 herring gulls, 100 great black-backed gulls, 20 grey-lag geese (*Anser anser*), 5 whooper swans (*Cygnus cygnus*), 4 tufted ducks (*Aythya fuligula*), 2 mallards (*Anas platyrhynchos*), 1 red-breasted merganser (*Mergus serrator*), 1 ring-necked duck (*Aythya collaris*), and 1 grey heron (*Ardea cinerea*) at Mill Loch, the freshwater body that had held the largest concentration of dead great skuas (Figure 3). Despite waterfowl being especially sensitive to HPAI [5], none of these birds showed any symptoms of HPAI infection (severe hyperextension and spasticity in the head and neck; opisthotonos), and no freshly dead birds were in that area, suggesting that the virus levels were too low to infect these waterfowl. Elsewhere on the island, there were large numbers of migrant birds, including thousands of redwings (*Turdus iliacus*); hundreds of fieldfares (*Turdus pilaris*), snow buntings (*Plectrophenax nivalis*), and meadow pipits (*Anthus pratensis*); smaller numbers of many other small passerines; and hundreds of shorebirds of various species. There were also several hundred herring gulls and great black-backed gulls resting on the clifftops and headlands. Resident starlings (*Sturnus vulgaris*), common ravens (*Corvus corax*), and hooded crows (*Corvus cornix*) scavenged across the island. Only three freshly dead birds were found—a single great black-backed gull that had swallowed a longline fishing hook and apparently died from that injury, and two redwings that were severely emaciated and appeared to have died of starvation during their migration. In February 2023, there was a period of exceptionally cold weather with heavy snow, which is unusual in Shetland. Several grey-lag geese at Foula died. Those birds were severely emaciated and their deaths were apparently due to being unable to feed because of the snow. None showed any of the symptoms of HPAI. It is, perhaps, surprising that there was no evidence of any birds being affected by HPAI after the outbreak in great skuas ended in August 2022 with the departure of the remaining surviving birds to their wintering areas, but this further suggests that HPAIV was probably not present in infective amounts after August 2022.

This small study suggests that removing rotting carcasses during the nonbreeding season would be unlikely to reduce the risks of avian influenza reappearing. The unexpected contrast between these results and the in situ study in Alaska [4] could be a result of the high rainfall at Shetland resulting in the virus being washed away. Rainfall in Shetland was >100 mm in August and in September 2022 [18]. Nevertheless, experimental evidence has shown that water represents a good matrix for virus survival and the infection of Galliformes, although such outcomes are likely to depend on the virus isolate used and dose administered (APHA, unpublished data). This leaves open the question of whether carcass removal early in an outbreak at a colony would reduce further transmission. It seems likely that removal of infected carcasses immediately after the death of the birds may reduce risk of spread of the infection. A more extensive assessment of water samples might be more informative, especially if sampling were able to occur during the outbreak event itself and at timepoints shortly after the apparent infection burnout.

In the absence of further data, we can only speculate as to which modes of transmission are most impactful on the maintenance of infection across different bird species in remote environments. However, it is possible to make some testable predictions based on knowledge of the ecology of this species. We know that diets of great skuas vary with the size of the colony, with more predation on other seabirds and scavenging around small colonies and more foraging on fish around larger colonies [19]. If scavenging on infected birds was the main route of infection for great skuas, then infections would be more likely to occur in smaller colonies. In contrast, if a migrant carries the infection from the wintering area and infects neighbours at the colony, then the largest colonies would be at greatest risk because more birds would mean more possibility of an infected individual returning to the colony and starting a new outbreak. An analysis of the infections that occur in future at different sizes of the colony, and possibly the creation of phylogenies based on virus sequencing to identify likely transmission events between colonies and between nonbreeding areas and breeding areas may help to identify the key routes involved. This would help to identify likely mitigation measures to reduce the impact on seabirds of high conservation concern. As the HPAI outbreak among great skuas in 2021 and 2022 may have killed about half of the entire adult population of this globally scarce species [3,7], there is a pressing need for further work on these issues.

In hindsight, our water sampling in October was too late after the peak of infection in June−July to monitor decline in virus levels. We should have started water sampling at the peak of the infection, but that was not achieved because the outbreak of HPAI in seabirds was an unexpected and novel event, arranging logistics took time, and evidence suggested that the virus would survive in the water for some months. There is an urgent need to plan for more detailed environmental sampling at seabird colonies during and immediately after future outbreaks, as these seem likely to occur in 2023 and beyond. Future planning can learn lessons from our experience reported here.

## Figures and Tables

**Table 1:** Location and date of samples tested by APHA from great skua carcasses sampled during the mass mortality in May−July 2022. Sample types were oropharyngeal swabs (OP) or cloacal swabs ©. The M gene was tested following Nagy et al. (2021) [14], High Path H5 was tested following James et al. (2022) [15], and N1 was tested following Payungporn et al. (2006) [16] and Slomka et al. (2007) [17].

Submission Number	Sample Type (OP/C)	Species	Location	Date Collected	M Gene	High Path H5	N1	Interpretation
AV002432-22	OP	Skua	Orkney	17 May 2022	+ve	+ve	+ve	HPAIV H5N1
	C				-ve	Not tested	Not tested	
AV002560-22	OP	Great Skua	Outer Hebrides	7 May 2022	+ve	+ve	+ve	HPAIV H5N1
	C				+ve	+ve	-ve	
AV002560-22	OP	Great Skua	Outer Hebrides	7 May 2022	+ve	+ve	+ve	HPAIV H5N1
	C				+ve	+ve	+ve	
AV002559-22	OP	Great Skua	Outer Hebrides	23 May 2022	+ve	+ve	+ve	HPAIV H5N1
	C				+ve	+ve	+ve	
AV002573-22	OP	Great Skua	Orkney	26 May 2022	+ve	+ve	+ve	HPAIV H5N1
AV002573-22	OP	Great Skua	Orkney	26 May 2022	+ve	+ve	+ve	HPAIV H5N1
	C				-ve	Not tested	Not tested	
AV002981-22	OP	Skua	Sutherland	June 2022	+ve	+ve	+ve	HPAIV H5N1
	C				+ve	+ve	+ve	
AV003180-22	OP	Great Skua	Foula, Shetland	11 July 2022	+ve	+ve	+ve	HPAIV H5N1
	C				+ve	+ve	+ve	
AV003180-22	OP	Great Skua		11 July 2022	+ve	+ve	+ve	HPAIV H5N1
	C				+ve	+ve	+ve	
AV003180-22	OP	Great Skua		11 July 2022	+ve	+ve	+ve	HPAIV H5N1
	C				+ve	+ve	+ve	

## Data Availability

Full details of the outbreak at Foula, Shetland, can be obtained from reference [3]. APHA’s data on HPAIV in wild birds are available from Bird flu (avian influenza): cases in wild birds—GOV.UK (www.gov.uk). Weather data are freely available from the online UK Met Office weather hub Met Office Weather DataHub-Met Office.
